# Composite media mixing Bragg and local resonances for highly attenuating and broad bandgaps

**DOI:** 10.1038/srep03240

**Published:** 2013-11-19

**Authors:** Nadège Kaina, Mathias Fink, Geoffroy Lerosey

**Affiliations:** 1 Institut Langevin, ESPCI ParisTech & CNRS UMR 7587, 1 rue Jussieu, 75005 Paris, France

## Abstract

In this article, we investigate composite media which present both a local resonance and a periodic structure. We numerically and experimentally consider the case of a very academic and simplified system that is a quasi-one dimensional split ring resonator medium. We modify its periodicity to shift the position of the Bragg bandgap relative to the local resonance one. We observe that for a well-chosen lattice constant, the local resonance frequency matches the Bragg frequency thus opening a single bandgap which is at the same time very wide and strongly attenuating. We explain this interesting phenomenon by the dispersive nature of the unit cell of the medium, using an analogy with the concept of white light cavities. Our results provide new ways to design wide and efficient bandgap materials.

There exist in nature numerous materials that prevent light from propagating, leading to various and sometimes amazing visual effects such as iridescence. Some of them, the so-called natural photonic structures, can be observed in the fauna, the flora or even in inorganic materials like opals^1^,^2^. Their properties are now understood to originate from their nanostructure that often presents periodic modulations. Their man-made counterparts, composite materials known as photonic/phononic crystals, present as well a wavelength-scaled periodic modulation of their optical^3^, elastic^4^,^5^ or acoustic^6^,^7^ properties. Analogous to atomic lattices^8^, they exhibit bandgaps^9^,^10^,^11^ within which the propagation of waves is prohibited. These bandgaps can originate from Mie resonances^12^ or from the structure. In the latter case, the associated gaps are named Bragg bandgaps, and they can be simply and schematically explained for a 1D structure by using the common Kronnig-Penney model of a periodic rectangular potential wells (*V*) chain (Fig. 1(a)). This schematic potential model, although appoximate, can fit all kind of scatterers like atoms (solid state physics) or index modulations (photonic crystals). When an incident wave propagates along the potential chain, it undergoes multiple scattering from the potential barriers, leading to interference effects between the different paths followed by the wave. For a frequency 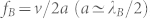, where *v* is the velocity of the wave in the medium and *a* is the lattice constant, those interferences are destructive, preventing the wave from propagating forward. At an interface, the medium acts like a mirror for waves and only an evanescent tail is allowed to penetrate. This Bragg bandgap is created with a midgap frequency *f_B_* set by the periodicity. In the case of periodic structures made out of strictly non resonant scatterers, the bandgap attenuation increases with the strength *V* of the potential barrier^8^ or equivalently the materials impedance mismatch in classical physics^3^, while its bandwidth decreases accordingly. Because the Bragg bandgap results from interference effects only, it is not affected by local modifications of the medium, which is of interest when one wants to control the flow of waves. Indeed, one can locally break the translational symmetry in photonic/phononic structures by introducing point^13^ or line defects^14^ whose resonances fall within the bandgap, which enables the waves to be either trapped or guided within the defects spatial extension roughly given by one period of the medium^3^.

Another kind of natural materials constitutes a very good mirror for electromagnetic waves: metals. They possess free electrons which reply in antiphase to an incoming electromagnetic wave, hence cancelling the latter on very short distances. In other words, metals present a negative permittivity and consequently a high reflectivity, below their characteristic plasma frequency *ω_p_*. To mimic this property but at macroscopic scales, some composite materials generally made out of subwavelength resonant inclusions have initially been proposed by Pendry^15^,^16^. Those so called metamaterials are organized at very subwavelength scales and are, consequently, often studied under an effective medium approximation. They have been mostly exploited for their high refractive indices^17^,^18^,^19^ which can be used for far-field subwavelength imaging or focusing purposes^20^,^21^,^22^,^23^,^24^ or for their negative effective properties^25^,^26^,^27^,^28^,^29^,^30^. As metals, metamaterials reflect waves within these negative effective property bands, which can hence be termed bandgaps. We have proved using a microscopic approach based on the transfer matrix formalism^31^ that the physics of locally resonant metamaterials presenting no near field interactions is solely driven by a Fano interference effect between the local resonance of the unit cell and the continuum of plane waves propagating in the medium^32^. The interferences occur because an incident wave impinging on a resonating unit cell can follow two paths, the non-scattered one and resonant one (Fig. 1(b)). If the unit cell resonates at *f*_0_, an incident plane wave at *f* > *f*_0_ results in a resonance response of the resonator in anti-phase(*ϕ* = −*π*) with the continuum exactly like a mechanical mass-spring that cannot follow a too fast excitation. For a monopolar resonator, this results in destructive interferences that give birth to a bandgap while cascading the resonators. This mechanism involves the coupling between the incident plane wave and a local resonance so that the bandgap of such materials can be interpreted as a hybridization bandgap^33^,^34^,^35^,^36^. Its position is solely given by the unit cell resonance frequency *f*_0_ which allows very subwavelength organizations as long as no near field interaction occurs directly between the unit cells. The gap extension and efficiency depend on the quality factor of the resonant unit cell and on the density of resonators. We have shown that in the limit of no near field coupling, the hybridization bandgap is understood to result from interferences. Consequenlty, analogous to photonic/phononic crystals, we have recently demonstrated that it is possible to locally modify such metamaterials to design very subwavelength components such as waveguides or cavities^32^. These components can be created in spatially disordered metamaterials as well^37^ because the hybridization bandgap originates from the resonance only and not from the periodicity.

From this brief review of composite materials, one naturally wonders what happens for materials presenting both a periodicity and a subwavelength resonant unit cell. It is known^35^,^38^,^39^,^40^,^41^,^42^, that those systems present, as expected, both bandgaps in their spectrum around *f*_0_ and *f_B_*. These can be independently shifted by changing either the frequency of the constitutive unit cell or the periodicity *a* and an overlap between the Bragg and hybridization bandgaps has been experimentally observed in phononic crystals made out of nylon rods^39^. Those bandgap materials are often studied in terms of their dispersion relation and group velocity. More precisely, while the Bragg bandgap presents a positive group velocity, the hybridization bandgap has a negative one, a signature of the materials negative effective properties^35^,^39^,^43^.

Here we go beyond these works by studying the interaction mechanism between the local and the Bragg resonances. To do so we investigate a hybrid photonic crystal/metamaterial where *f*_0_ and *f_B_* perfectly match (Fig. 1(c)) by adjusting the lattice constant to 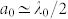, where *λ*_0_ is the resonance wavelength associated with *f*_0_. Those systems have been studied in solid state physics and named resonant Bragg reflectors, for instance with Bragg spaced multiple quantum wells^44^,^45^ and more recently a plasmonic equivalent has been proposed^46^. Yet those latter works are based on a quantum approach in which excitons embedded in a periodic potential well lattice^44^,^45^ or plasmonic resonances embedded in a dielectric medium^46^ radiate a Lorentzian-shaped linewidth. In this framework, it has been theoretically proved that they can give rise, depending on the number of periods, either to a superradiant mode or to a photonic bandgap. In this paper, we demonstrate an experimental realization of resonant Bragg reflector in the microwave domain which allows us, contrary those previous works^44^,^45^,^46^, to probe locally the electromagnetic field in the composite medium. This allows us to leave out the quantum formalism used in these references and to give a very simple explanation through a classical wave approach. We stress here that in the systems studied in this paper, both Bragg and hybridization effects arise from the same element that is the resonator: this is why it can be called a resonant Bragg structure. It should not be mistaken with another way of mixing Bragg and resonances that has also been widely studied in solid state physics and which consists in filling a pre-existent periodic structure from which the Bragg interferences arise with a polarizable medium, giving raise to so called Braggoritons^47^,^48^.

We first use the formalism developed in^32^ and investigate the theoretical two dimensional case of a linear array of split rings placed in a single mode waveguide. We observe that for specific periodicities the hybridization and Bragg bandgaps mix into a single one which presents at the same time a very large attenuation and a very large bandwidth. We experimentally verify this property using a quasi-one dimensional composite medium consisting of split-ring resonators coupled with a transmission line. By mapping the near field of the medium, we prove that the very wide and strongly attenuating bandgap obtained results from Bragg scattering off the resonant unit cells of the medium. We finally interpret this result as a consequence of the dispersive nature of the unit cell, in analogy with the concept of White Light Cavities (WLC)^49^,^50^.

## Results

### Numerical results

We start our study by simplifying the problem as much as possible and to do so we consider a quasi-1D system consisting of a linear chain of “atoms” enclosed in a single mode waveguide. The “atoms” are split ring resonators (SRR) which are illuminated by a transverse magnetic field and whose dimensions, noted in Fig. 2(a), are *L* = 0.12 m, *e* = 0.01 m and *e_g_* = 0.001 m. Those resonators are placed in a waveguide made out of perfect electric conductor walls whose height is much smaller than half a wavelength, hence ensuring that it is single mode. We simulate with a finite element method a single SRR placed in the center of this waveguide which is several wavelengths long. Its complex transmission *T* is plotted in Fig. 2(b). In our previously published microscopic theory^32^ based on the transfer matrix formalism^31^, we proved that the transmission of a single resonator is sufficient to numerically calculate the dispersion relation *k*(*a*, *f*) of an infinite 1D chain of resonators (Fig. 2(c)). This can be done using the following formula as long as the near field interactions between the unit cells are negligible, which is the case here for any period *a*, as demonstrated in^32^: 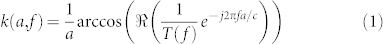
where *T*(*f*) is the complex transmission coefficient, *f* is the frequency, *a* is the periodicity of the infinite chain and c is the speed of light (Fig. 2(c)). The real part of *k* obtained in (1) gives the propagating modes of the system, while its imaginary part corresponds to the attenuation, which in this lossless system relates only to the bandgaps. This is the quantity that is of interest for our study. To understand how the lattice constant influences the dispersion relation of a locally resonant quasi-1D metamaterial, we compute *k* for a set of lattice constants *a* using (1). We display its imaginary part as a function of the frequency and the periodicity (Fig. 2(d)). From Fig. 2(d), we clearly observe that the dispersion displays a very different behavior depending on the periodicity. The bandgaps correspond to the yellow-orange colored parts of the map while the purely propagative parts of the spectrum are the black ones. For most periodicities, the system presents two bandgaps. The first one starts at *f*_0_, is very asymmetric with a strong attenuation, it corresponds to the hybridization bandgap^33^,^34^,^35^. The second one appears at positions which depend on *a* (white lines), its shape is symmetric and its attenuation is limited, it is related to Bragg scattering^3^. However, we note some interesting periodicities where the spectrum displays a single bandgap (red dashed lines) that is much broader than the simple Bragg and hybridization bandgaps. It corresponds to the points where the Bragg frequencies match the local resonance one. This bandgap is very interesting since it is at the same time very broad and attenuates strongly the waves, which is unusual if compared to Bragg bandgaps of non-resonant scatterers. It is expected to happen for the specific periodicities *a*_0_ = *nλ*_0_/2 (where n is an integer and *λ*0 is the wavelength in the empty waveguide) but we observe a slight shift from this theoretical value due to the finite quality factor of one resonator. We expect the theoretical value to be reached for very high *Q* resonators. We note that as expected intuitively, far from the resonance *f*_0_, the classical Bragg scattering is not affected since the resonators act like non-dispersive scatterers. To experimentally observe and investigate the very interesting properties of the hybrid Bragg/hybridization bandgap enlightened by the simulation results, we realize an experiment that mimics as much as possible this simplified theoretical system.

### Experimental results

We want to study a quasi-1D medium made out of split rings which resembles the one studied before and hence we opt for samples printed on an epoxy substrate sitting on a ground plane. The single mode waveguide is experimentally realized by a 50 Ω microstrip transmission line (length 250 mm, width 3 mm) which is near field coupled to SRRs (*L* = 4.4 mm, *e_g_* = 1 mm, *e* = 0.9 mm). The coupling occurs because the magnetic field lines created by the transmission line cross the SRR in the out-of-plane dimension, providing the good symmetry for the SRR to be excited. The strength of the coupling Γ can be tuned by adjusting the distance between the line and the resonators. We choose the latter (*d* = 0.5 mm) so that the SRRs couple much faster to the line than to free space, hence mitigating radiation losses. Thus, the relaxation of the SRRs occurs mostly through the microstrip. We stress here that the split rings have no direct capacitive or inductive interaction since they are distant enough. This setup ressembles Coupled Resonator Optical Waveguides (CROWS)^51^,^52^ except for the fact that the resonators are not very high *Q* ones and are coupled through a transmission line rather than evanescently. The transmission line is terminated on both sides with SMA type 50 Ω connectors, and we measure its transmission coefficient *S*_12_ by connecting it to a network analyzer. We first measure the transmission of a sample which contains a single SRR (Fig. 3(a)) and find a resonance frequency *f*_0_ = 7.31 GHz that depends on the geometrical dimensions of the SRR. This resonance presents a rather symmetric profile because the set-up enables a symmetric coupling between the SRRs and the incident wave. We stress here that the strength of the asymmetry of the Fano coupling depends on the geometry of the system^32^.

We then experimentally study one dimensional chains of 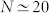 SRRs depending on the periodicity. We fabricate and measure nine samples differing only by the lattice constant. We display here the results for five of them (*a* = 8 mm, *a* = 10.3 mm, *a* = 11.1 mm, *a* = 11.7 mm and *a* = 15 mm). For all samples except for the middle one (Fig. 3(b)–(c)–(e)–(f)), we clearly observe two bandgaps. This is expected since the systems present both a local resonance through the SRR and a periodicity. The hybridization bandgap (in orange) is asymmetric and remains around the resonance frequency *f*_0_ while varying the lattice constant, consistent with our expectations. The second bandgap (in green) is relatively symmetric and is significantly shifted with the periodicity. It corresponds to the expected Bragg bandgap. Those two bandgaps arise from completely different phenomena and display different characteristics, mostly in terms of dispersion and group velocity^39^,^43^.

From Fig. 3(b)–(c)–(d), we see that the efficiency of the Bragg bandgap varies with the periodicity. This can be explained by the fact that the efficiency depends on the scattering cross section of the resonators, which is a dispersive quantity that decreases as we drift from *f*_0_. We now focus on the third sample (Fig. 3(d)). It is the hybrid photonic crystal/metamaterial one, for which the Bragg frequency matches *f*_0_. Instead of the two bandgaps from hybridization and Bragg interferences, respectively asymmetric and symmetric, we observe a single almost symmetric, broad and very efficient bandgap. It presents a bandwidth at −3 dB of around 1.2 GHz, that is, 17% of the central frequency. This experimentally confirms the behavior observed in the simulation (Fig. 2(d)). In contrast with the numerical simulation, this bandgap presents an almost symmetric shape that arises from the likely symmetric transmission coefficient of a single SRR. To understand the underlying physics of this specific broad and efficient bandgap, we perform further experiments on the sample of Fig 3(d).

From now on, we focus on the sample whose period *a* is almost the value *a*_0_ that opens the single bandgap. In order to better understand the nature of this special sample, we seek for the field distribution above the line and the resonators. To that end, we map the magnetic near field above the sample by means of a very small hand-made loop probe placed 1 mm above it and mounted on a 2D translation stage. For each spatial position, we measure the *S*_12_ coefficient between the incident field that feeds the transmission line and the loop with a network analyzer. The microstrip transmission line is terminated by a 50 Ω load to avoid any reflection at the end of the line. The logarithmic map of the H-field (Fig. 4(a)) is given for three frequencies within the single bandgap (dashed lines in Fig. 3(d)). On each scan, we clearly see two regions where the transmitted field is maximal. The first one corresponds to the transmission line and the second one to the SRR array (see photo). On the latter, the field is maximal above each SRR, thus confirming their resonant behavior. As a signature of the bandgap, we observe a clear decrease of the field along the line from the input feed point (*x* = 0) to the output point (*x* = *L*). The faster decrease occurs naturally for *f*_0_, where the bandgap is the most efficient (Fig. 4(b)).

## Discussion

**Figure 1:**
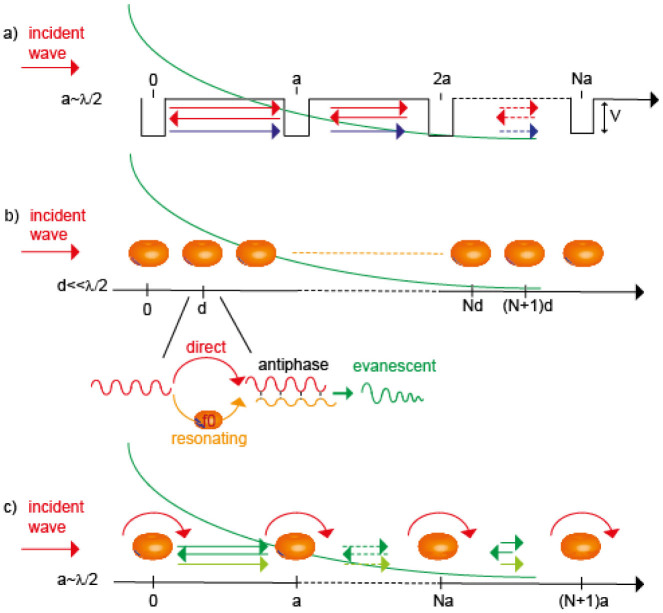
(a) Bragg interferences through a one dimensional potential wells chain of periodicity *a*. Red and blue arrows stand for waves interfering destructively.(b) Hybridization interferences through one dimensional resonators (*f*_0_) chain with periodicity *d* ≪ *λ*. Inset shows the destructive interference mechanism between the direct and resonating paths for one resonator. (c) Hybrid Bragg/Hybridization chain of resonators (*f*_0_) separated by a periodicity *a*_0_ = *λ*_0_/2. Both Bragg scattering (green arrows) and resonance (red arrows) take part to the resulting evanescent forward wave (green).

**Figure 2:**
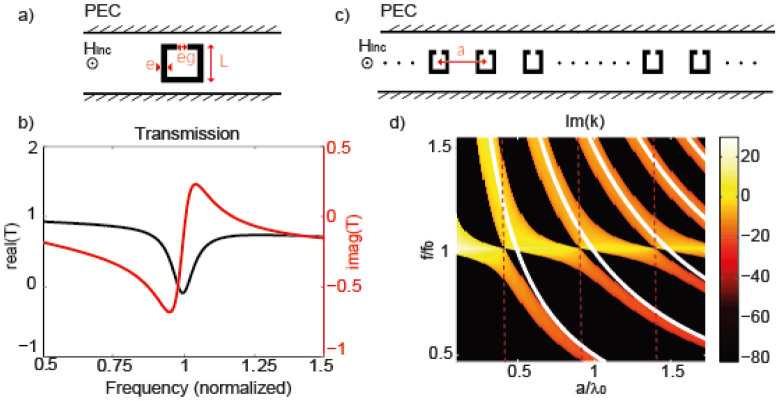
Simulated split ring resonator within PEC waveguide (a) and corresponding transmission coefficient (b). Infinite chain of the same SRR unit cell with variable periodicity *a* (c) and corresponding dispersion relation as a function of *a* and the frequency (d).

**Figure 3:**
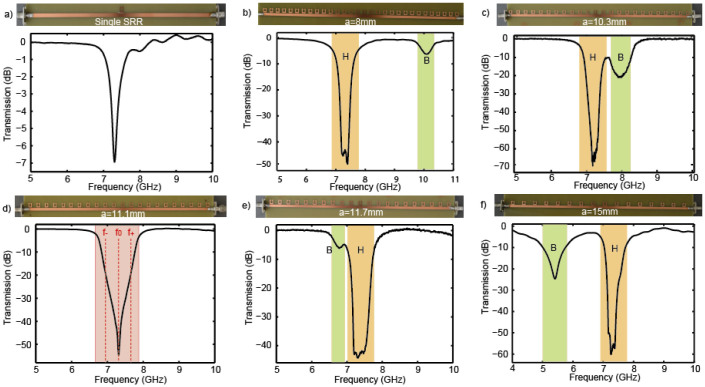
Photos of the samples and normalized logarithmic transmission through the transmission line for a single SRR (a), a chain of 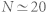 SRRs with periodicity *a* = 8 mm (b), 10.3 mm (c) 11.1 mm (d), 11.7 mm (e) and 15 mm (f). Bragg bandgap (green), Hybridization bandgap (orange), mixed bandgap (red).

We further show an enlargement of the map on a couple of SRRs and its corresponding part of the transmission line at *f*_0_ in Fig. 5(a). We note that the SRR and the transmission line are both excited. Interestingly, the maximum of the field above the microstrip is shifted from the SRR and occurs somewhere between the two resonators. The profiles of the H-field above the SRR (black) and transmission line (red) (Fig. 5(b)) show that the field above the TL seems to be trapped within two SRRs, as it would be in a cavity made out of two potential barriers, except that in our case, the potential *V* is dispersive due to the resonant behavior of the SRR. This result enables us to explain the physics of this interesting phenomenon while comparing it to the concept of WLC^50^. It is well known that cavities present resonant frequencies that depend on their length. The resonance occurs for frequencies that lead to a round trip that ensures a 2*π* phase accumulation within the cavity. The cavity bandwidth, that is the frequency domain around the resonance on which the constructive interferences occur, is limited by the strength of the potential *V* of the walls of the cavity. Indeed, the higher *V*, the higher the number of round trips of the waves in the cavity and thus the larger the number of wave periods interfering. This makes the interference condition very sensitive to any phase difference between the multiply scattered waves and hence leads to a smaller bandwidth, or equivalently to a higher *Q*-factor. The concept of WLC has been proposed a few years ago to overcome this limitation^49^,^50^. It enables the bandwidth of the cavity to be significantly increased. This can be achieved by filling the latter with an appropriate dispersive medium or by using chirped gratings. Owing to their dispersive nature, both systems bring an extra phase when the wave travels in the cavity which maintains the constructive interference condition way beyond the purely geometric resonance of normal cavities when the frequency of the travelling wave is shifted off the resonance. In analogy with cavities, photonic crystals based on high potential barriers present bandgaps which are very efficient, but are accordingly narrow. In our system, the spectrum exhibits a single a much broader bandgap compared to the Bragg bandgap that would be opened in an equivalently efficient but non resonant system. That means that the destructive interference between the directly transmitted wave and the wave transmitted after a round trip travel (Fig. 1(a)) can be extended to a large part of the spectrum, far beyond the geometric Bragg condition. Indeed, equivalently to WLC, an extra phase is brought by the resonators which present a dispersive response. The total phase Φ of a round trip travel includes the travelling phase Φ*_prop_* in the non-dispersive medium and the phase resulting from twice the reflection off the resonators, which is 2*Φ*_r_* where Φ*_r_* is the phase of the reflection coefficient *R* of one resonator. Upon each reflection the wave is solely coming from the resonator, and hence it displays the same phase shift as the latter around the resonance, that is that it goes from 0 to −*π* around the resonance, which compensates for the propagating phase as shown in Fig. 5(d). Naturally, the lower the Q-factor of the resonator, the larger the frequency range where this effect occurs. The physics underlying the concept of WLC is hence exactly the same that explains the properties of locally resonant metamaterials organized at the Bragg period. The latter can be seen as simple Bragg bandgaps with dispersive scatterers. The difference lies in the fact that in our case the compensating dispersion is brought by the scattering walls and not by filling the cavity with a dispersive medium or by using chirped gratings and that a destructive (bandgap) and not constructive (cavity) interference condition is maintained. It is worth noting that the concept studied here permits a new type of WLC to be designed as well. Indeed, using simply two resonators instead of a linear array as here results in a Fabry-Perot with dispersive mirrors.

Finally we want to point out that this approach is valid as long as the resonators present no near field coupling, which should be the case for any resonator constituting of a metamaterial when considering periods around *λ*_0_/2 involved here. It is clear for instance that using lower Q-factor resonators will result in slightly less efficient yet even much wider bandgaps. Even though we only considered the simplest case of a one dimensional medium, the concepts in our approach and the main results should be valid in 2D and 3D. Yet the physics associated should be even more interesting since for those dimensionalities both the unit cell symmetry of the medium and the scattering cross-section of the resonators will play an important role in the dispersive properties of the crystals.

## Methods

The numerical results were obtained using the finite elements method software Comsol Multiphysics. We simulated using the Transverse Magnetic solver only one split ring resonator in a single mode perfect electric conductor waveguide. The complex transmission coefficient of a single resonator corrected by the propagation term was obtained by normalizing the response of a single resonator in a waveguide by that of an empty waveguide. The complex propagation constant of an infinite medium was then calculated using Equation 1 which was derived in the Supplementary Information of^32^ using the transmission matrix approach.

**Figure 4:**
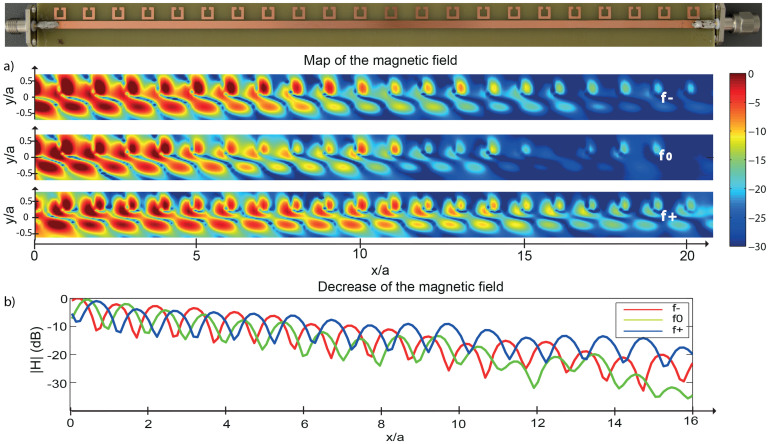
(a) Photo of the sample *a* = *a*_0_ along with the corresponding map of logarithmic H-field for *f*_−_ = 6.93 GHz (up), *f*_0_ = 7.31 GHz (middle) and *f*_+_ = 7.69 GHz (down). (b) profiles of the logarithmic decay of the H-field along the transmission line for the three frequencies.

**Figure 5:**
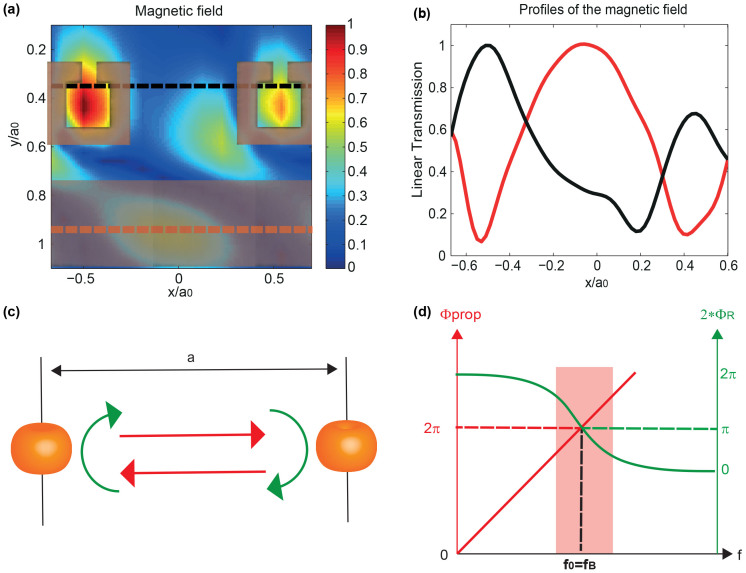
(a) Enlargement on two SRRs with superimposed line and SRR scheme for *f*_0_. Dashed lines correspond to the profiles. (b) Profile of linear field on the transmission line (red) and SRR line (black) between two SRRs. (c) Schematic view of the a cavity formed by two adjacent resonators. Red arrows stand for the propagation and the green arrows for the reflection on the resonators. (d) Schematic view of the propagating phase (red) and the resonating reflection phase (green) within a round-trip as a function of the frequency of the travelling wave. The red rectangle shows the frequency excursion for which the total phase sticks to *π*.

Concerning the expriments, all the samples were printed on epoxy substrates using standart circuit type lithography processes. SMA (SubMiniature version A) connectors were soldered at each side of the samples in order to plug them to the network analyzer or to an impedance matching load. The network analyser (Agilent N5230C PNA) was set to its maximum number of points, 20000, and we used a reasonable power output of 5 dBm. No averaging was used since the dynamic of the measurements was very good, and the sweeping time was set to 20 ms. All the transmission measurements were obtained by measuring the S parameters between the two ports of the network analyser. The near-field measurements of the sample at the Bragg condition were realized by matching it on one side using a 50 Ohms load while the other side was connected to port 1 of the analyser. The port 2 of the analyser was connected to a small magnetic near field probe, i.e., a 2 mm diameter loop realized using the core of a SMA coaxial cable. We performed 40 by 200 measurements using this small magnetic loop 1 mm on top of the sample measuring 5 cm by 20 cm. The maps of Figures 4 and 5 are obtained by filtering spatially the data to remove the high frequency spatial noise, and, oversampled in order to smooth it.

## Author Contributions

G.L. initiated and supervised the project, N.K. performed the simulations and experiments, G.L and N.K. analyzed the results and developed the theory, G.L., N.K. and M.F. discussed the results and wrote the manuscript.

## Supplementary Material

Supplementary Information Supplementary information
