# Supplementary material for: Composite media mixing Bragg and local resonances for highly attenuating and broad bandgaps
Source: Sci Rep. 2013 Nov 19;3:3240. doi: 10.1038/srep03240 (PMC3832873; doi:10.1038/srep03240)
Supplement: Supplementary Information [file srep03240-s1.pdf]

Supplementary Information for: Composite media mixing Bragg and local resonances for highly attenuating and broad bandgaps

Nadège Kaina, Mathias Fink, and Geoffroy Lerosey*
Institut Langevin, ESPCI ParisTech & CNRS UMR 7587,
1 rue Jussieu, 75005 Paris, France

1. The reflected phase

To support the assertion that the phase of the reflection coefficient is anomalous and goes from 0 to $-\pi$ at the resonance frequency of the SRR (upon which is based our phase compensation analysis), we extract the R coefficient in our COMSOL simulation (described in paragraph RESULT/Numerical results of the manuscript). To do so, we simulated the SRR within the waveguide as well as the waveguide alone as a reference. Both waveguides dimensions are L=5m and h=0.181m. The reflection coefficient is taken as the integral of the H field along the input height of the waveguide. The R coefficient from the SRR only can be extracted while subtracting the reference H field of the waveguide alone and multiplying it by a propagating factor, corresponding to the travelling distance between the input and the SRR. The phase of this so calculated R coefficient as well as the simulation setup are given in figure S1.

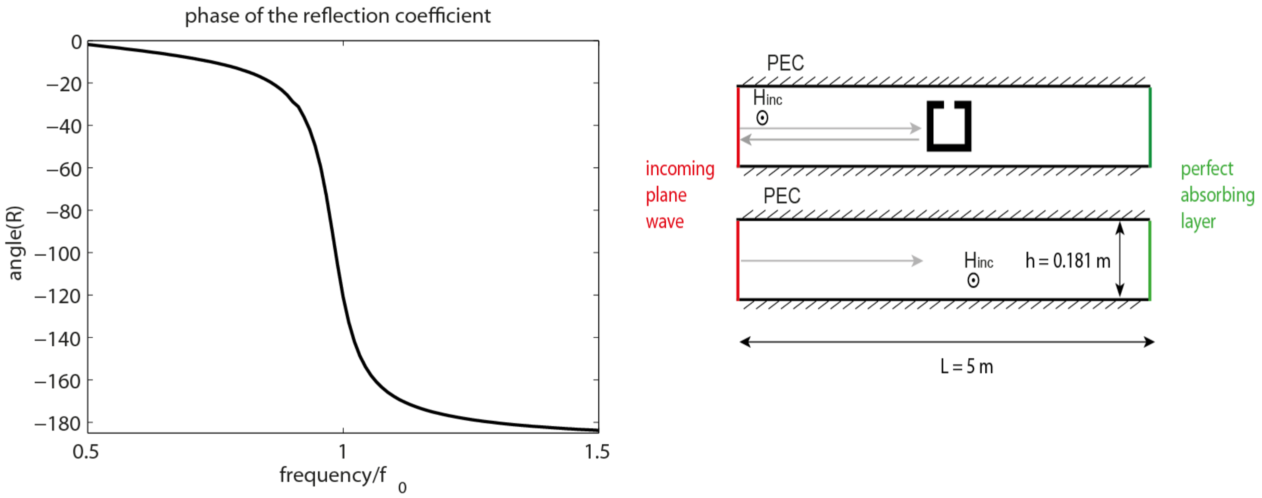

FIG S1: (Left) Phase of the simulated reflection coefficient (simulation parameters of the manuscript) in degrees as a function of the frequency. (Right) Simulation setup

2. Higher orders Bragg bandgap

In Fig.3 of the manuscript, we present the experimental spectra for a restricted frequency range varying from 4 or 5 to 10 or 11 GHz, depending on the periodicity. For those frequency ranges, we could not observe the second order of the hybridization bandgap or any higher orders of the Bragg

bandgaps. However, we performed the measurement over a total bandwidth that goes from 2 GHz to 12 GHz. This allows us to observe the second order Bragg bandgap for the largest periodicity (a=15mm, Fig.3(f)). The spectrum over the whole bandwidth is presented in Fig. S2. We clearly see that there is a bandgap around the expected frequency f_{B2} ~2*f_{B1}, and that, owing to the greater distance to the hybridization bandgap (and hence a lower polarizability of the SRR), the efficiency (as transmission depth of the bandgap) is much lower.

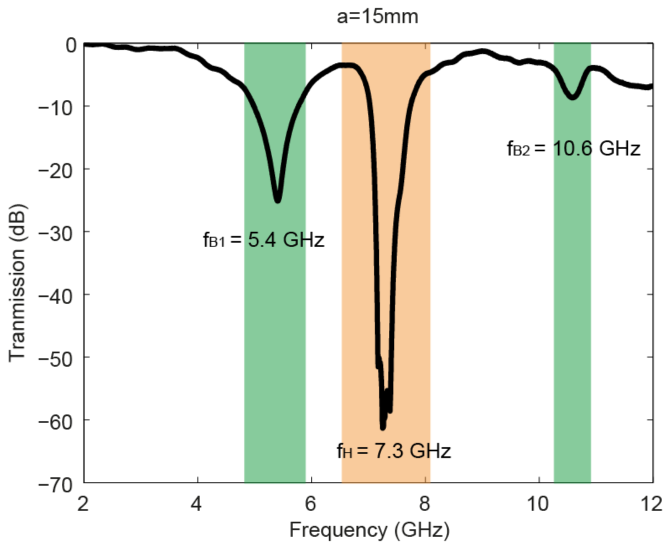

<u>FIG S2</u>: Transmission in dB through the transmission line for a chain a=15mm

3. Discussion on the periodicity parameter that opens the wide bandgap

a. The value of a0

In theory, the parameter a0 corresponding to the perfect match of the Bragg frequency and the resonance frequency f_0 should be $a_0=\lambda_0/2$, where λ_0 is the wavelength associated to f_0. However we observed in simulation that the wide and single bandgap opens for a parameter ‘a’ that is slightly shifted from a0. For example, in the simulation described in the manuscript, this parameter is a=0.411* λ_0. We performed simulations to understand where this deviation comes from. To do so, we varied the parameter e_g (the width of the SRR’s slit) that is linked to the quality factor of the SRR’s resonance (the smaller e_g, the greater Q) and extracted the parameter ‘a’ that opens the single bandgap. From Fig.S3, we observed that the greater Q is, the closer ‘a’ to ‘a_0’ is. This value of ‘a’ can be greater than 0.5* λ_0 depending on the kind of resonator and the geometry of the setup. In this case, ‘a’ would decrease as Q increases.

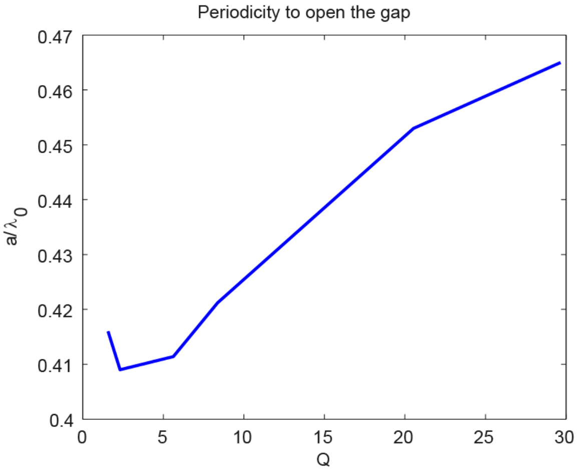

FIG S3: periodicity that opens the wide bandgap as a function of the quality factor of the resonance

In our simulation, the Q factor (due to radiative losses only since we introduced no source of dissipation) is around 5. In our experiment, the Q factor of a single SRR is around 30 (radiative and dissipation losses). This can explain why the parameter 'a' that opens the single bandgap is closer to 'a0' in our experiment (a=0.485* λ_0) than in the simulation.

b. The parameter range that opens the single bandgap

In the simulation of paragraph "Numerical Result", we observe that the parameter 'a' that opens the single bandgap is very singular. This is due to the fact that we introduced no dissipation sources in our simulation. Of course, in a real experiment such losses occurs and we did not expect the periodicity range to open the gap to be singular. We display in Fig.S4 the experimental value of 1-|T| (schematically corresponding to im(k)) as a function of the frequency and the periodicity 'a' for the 9 samples we fabricated. We see that we have a single bandgap for a large range of 'a', even if the resulting bandgap is not as symmetric as for the right 'a'. This is what is for example observed in figures 3(c) and (e) .

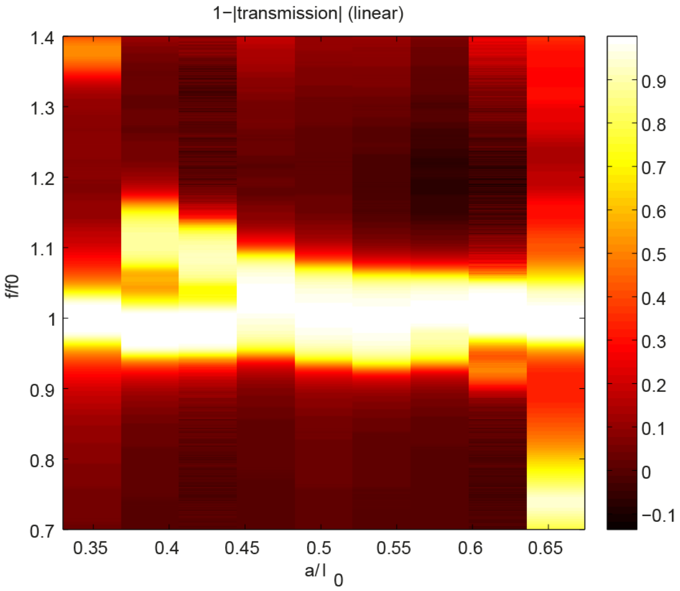

FIG S4: Map of the experimental 1-|S12| for 9 periodicities across the frequency range
